# Biophysical Interactions Underpin the Emergence of Information in the Genetic Code

**DOI:** 10.3390/life13051129

**Published:** 2023-05-04

**Authors:** Aaron Halpern, Lilly R. Bartsch, Kaan Ibrahim, Stuart A. Harrison, Minkoo Ahn, John Christodoulou, Nick Lane

**Affiliations:** 1 UCL Centre for Life’s Origins and Evolution (CLOE), Department of Genetics, Evolution and Environment, University College London, London WC1E 6BT, UK; 2 Department of Structural and Molecular Biology, Institute of Structural and Molecular Biology (ISMB), University College London, London WC1E 6BT, UK

**Keywords:** origin of life, genetic code, biophysical interactions, hydrophobicity, anticodon, molecular dynamics, NMR

## Abstract

The genetic code conceals a ‘code within the codons’, which hints at biophysical interactions between amino acids and their cognate nucleotides. Yet, research over decades has failed to corroborate systematic biophysical interactions across the code. Using molecular dynamics simulations and NMR, we have analysed interactions between the 20 standard proteinogenic amino acids and 4 RNA mononucleotides in 3 charge states. Our simulations show that 50% of amino acids bind best with their anticodonic middle base in the −1 charge state common to the backbone of RNA, while 95% of amino acids interact most strongly with at least 1 of their codonic or anticodonic bases. Preference for the cognate anticodonic middle base was greater than 99% of randomised assignments. We verify a selection of our results using NMR, and highlight challenges with both techniques for interrogating large numbers of weak interactions. Finally, we extend our simulations to a range of amino acids and dinucleotides, and corroborate similar preferences for cognate nucleotides. Despite some discrepancies between the predicted patterns and those observed in biology, the existence of weak stereochemical interactions means that random RNA sequences could template non-random peptides. This offers a compelling explanation for the emergence of genetic information in biology.

## 1. Introduction

“The whole case here rests upon the demonstration that codon-amino acid pairing interactions exist and that the codon assignments in some way reflect these interactions… all-or-none specificities are not required for such interactions to determine the form of the codon catalog, either a general form or one specified down to the very last detail. All that is required here is that a sufficient number of slight preferences be shown.” Carl Woese, 1969.

The origin of the genetic code and the emergence of biological information is a notoriously elusive question. Even on its discovery, it was clear there are non-random patterns in the code [1,2,3]. These patterns loosely correspond to the biosynthetic precursors of the amino acids encoded [4,5,6,7], the hydrophobicity of those amino acids [8,9], and less clearly, their size [4,10], all of which point to some kind of direct biophysical interactions. Nonetheless, as suspected by Woese as early as 1969, ‘all-or-none preferences’ do not exist [1,11]. Woese argued that a large number of slight preferences (weak and not highly specific) could still have played a strong role in fixing the codon assignments [11]. Yet, this prescient argument was displaced by Crick’s adaptor hypothesis, which highlighted the lack of direct interactions between amino acids and either the codon or anticodon in tRNA [12,13]. Crick’s position was unequivocal: since neither evidence for such interactions nor a reasonable model for them existed, the interactions themselves did not exist under any circumstances [9,14]. While others have continued to pursue the idea that direct stereochemical interactions underpin the code, even the most promising experiments and simulations over the following half century [15,16,17,18,19,20,21,22,23,24,25,26] did little to dispel the prevailing scepticism [27,28,29]. 

A second theme of Woese’s early ideas on the code has also been largely neglected since the rise of the RNA world hypothesis. Woese noted that the main ideas on the optimisation of the code implicitly assumed selection at the level of the cell, whether through minimising the effects of mutations [30,31], constraining ambiguity [32,33,34,35], expanding an early amino acid vocabulary [13], or finetuning weak biophysical preferences into the all-or-none assignments seen today [9,11,35]. In contrast, the RNA world hypothesis advocated selection at the level of the gene. Beginning with another foundational paper in 1968, Orgel questioned how far complexity could emerge in a peptide world versus an RNA world [36]. He concluded that only the simple copying of RNA templates could account for the emergence of natural selection, and so focused attention on the replication of RNA as a unit of selection [36]. By the early 1980s, the discovery of ribozymes gave the RNA world a sense of concrete reality [37,38,39,40]. Orgel’s influential scenario held that “the very first replicators were ‘naked genes’ adsorbed on the surface of mineral particles”, and later on, “impermeable membrane caps were ‘invented’ by the genetic system as it became metabolically competent [41].” While these ideas do not rule out the emergence of the genetic code in cells, selection at the level of RNA is proposed to generate a set of functional RNA catalysts that sustain exponential growth in a prebiotic environment [40]. Metabolism emerged with ‘RNA cofactors’, such as NAD, and the first proteins performed the same reactions as ribozymes, but more effectively, thereby eventually displacing RNA [40,42]. Neither the code nor cells are emphasised as critical early steps. 

There is no doubt that RNA played a determining role in the early translational system and the emergence of the code [39,43]. Yet, the idea that RNA ‘invented metabolism’, though dominant, has not been expansively developed. Attention has instead focused on the difficult problem of RNA replication [42,44,45] and escaping the tendency to select for replication speed, often leading to parasitic collapse [46,47,48,49,50]. Producing stable longer-chain RNAs has been an end in itself [51], on the assumption that natural selection can then drive the emergence of metabolism, ‘bit by bit’ [52]. Yet, this scenario overlooks the complexity of metabolic pathways, the known endpoint of evolution. While cofactors probably played an important role, by no means do they catalyse every step. If RNA encoded ribozymes or enzymes that each catalysed individual steps, introducing them one at a time would have no benefit if the rest of the pathway was missing [53,54,55]. Building metabolic pathways step by step from one end or the other lowers the combinatorial odds, but requires that all intermediates be stable, useful, and available [56,57,58], which is certainly not the case for pathways such as purine synthesis. The problem is not simply combinatorial. As observed by Walker and Davies, “biological information has an additional quality which may roughly be called ‘functionality’—or ‘contextuality’—that sets it apart from a collection of mere bits as characterised by its Shannon information content.” [59] The simplest solution to this near-intractable problem is to assume that the core of metabolism is thermodynamically and kinetically favoured in a propitious environment [60,61,62], so the first RNA genes only had to enhance flux through the protometabolic network [63]. Yet, that presumes a lot from prebiotic chemistry, to the point that Orgel memorably dismissed it as ‘an appeal to magic’ [64].

Experimental work over the last decade has now weakened Orgel’s position. Starting from CO_2_ and H_2_—the autotrophic core of all life—much of intermediary metabolism is not only thermodynamically favoured, but occurs spontaneously following the universally conserved biochemical pathways. Mineral catalysts [65,66] or proton gradients across inorganic barriers [67] can drive CO_2_ fixation, directly generating the carboxylic acid intermediates of the acetyl CoA pathway [68] and reverse Krebs cycle [69,70]. From these universal precursors, α-amino acids [70,71,72,73], acetyl phosphate [74], sugars [75,76,77,78], and some nucleobases [79] have been formed via pathways that prefigure metabolism in the absence of genes and enzymes. While much still needs to be done, the concept of a spontaneous protometabolism is no longer an appeal to magic. Going further, modelling shows that catalytic positive feedbacks from nucleotide cofactors could drive flux through protometabolism, giving rise to autotrophic protocells growing from CO_2_ and H_2_ [63]. If RNA polymerisation could occur within these replicating protocells, the emergence of the genetic code through weak biophysical interactions between amino acids and cognate bases could solve the RNA world problem along the lines postulated by Woese [11].

Reexamining patterns in the code from the standpoint of autotrophic protometabolism is little short of revelatory. Harrison et al. have shown that the base at the first position of the codon corresponds to the distance from CO_2_ fixation, following the universal metabolic map [63,80]. Amino acids encoded by a G at the first position of the codon are usually the closest to CO_2_ fixation, followed by A, then C and U, which might suggest a purine-rich early metabolism [80]. Given that most nucleotide cofactors are derived from purines, including NAD, FAD, CoA, ATP, folates, and pterins, the idea of a purine-rich early metabolism is consistent with cofactor-catalysed positive feedbacks. When structured according to the base at the first position of the codon, there is a much stronger relationship between the hydrophobicity of the amino acid and the base at the second position of the anticodon—in other words, the correlation is stronger for earlier amino acids (closer to CO_2_ fixation) than for later amino acids [80]. Finally, the patterns of redundancy across the code are far from random, but are governed by rules pertaining partly to the size of the amino acid and adjoining bases [80]. These patterns predict weak biophysical interactions between amino acids and cognate bases. As noted, while there are tantalising hints that such interactions do exist [15,16,17,18,19,20,21,22,23,24,25,26], they have not yet been systematically demonstrated across the full code [27,28]. Here, we have taken a novel approach, using molecular dynamics simulations to analyse the forces acting between atoms, which has allowed us to revisit the interactions between the 20 standard proteinogenic amino acids and 4 RNA mono-nucleotides in 3 distinct charge states. We find that half of the amino acids do bind best with their anticodonic middle base in the −1 charge state common to the backbone of RNA (which is greater than 99% of randomised assignments), while 95% of amino acids interact most strongly with at least 1 of their cognate codonic or anticodonic bases. We verify a selection of our results using NMR. Our results corroborate Woese’s proposals from more than half a century ago, and offer a compelling framework for the emergence of genetic information in biology.

## 2. Methods

### 2.1. Molecular Dynamics Pipeline

Mol2 files representing the 20 standard proteinogenic L-amino acids in a zwitterionic state and with protonation states representative of pH 7 were produced in Avogadro [81]. Where used, dinucleotides were simulated in the NpN format. For each of AMP, CMP, GMP, and UMP, 3 files were also produced with the phosphate at either a −2, −1, or neutral charge. These files were then uploaded to CHARMM-GUI’s Ligand Reader and Modeller [82,83], which output psf, crd, prm, and rtf files for the molecules. These were passed to CHARMM-GUI’s Multicomponent Assembler [84] to produce the input files for the MD simulations. Each system contained 10 copies of an amino acid and 1 nucleotide in order to increase the frequency of collisions. A 40 Ångstrom periodic box was used, with water as the solvent. For charge neutralisation, 150 mM MgCl_2_ was added using Monte Carlo ion placement, resulting in a variable number of ions, depending on the charge states of the monophosphate and amino acid. Default PME parameters were used. The temperature was set at 25 °C using an NVT ensemble. 

The input files were then used as starting points for the simulations using NAMD 2.0 [85] and the CHARMM-36m forcefield [84], which uses a modified version of the TIP3P water model. Simulations were minimised and equilibrated for 10,000 and 125,000 timesteps, respectively, and run for 48 h using 2 MPI cores 100 times in parallel on UCL’s myriad cluster, with randomised initial velocities in each parallel run. This produced total simulation times of approximately 1.5 microseconds. 

Output trajectories were extracted in MATLAB R2020b using MDtoolbox [86]. The Euclidian distance between the nucleotide and the closest amino acid was calculated, as determined by the distance between the closest atom of each molecule. Binding was approximated by time spent within a 5 Å threshold. This value was chosen qualitatively as a single threshold covering the many varied binding modes observed between the large range of molecules. Uncertainty was calculated by treating the 100 parallel runs as individual experiments and bootstrap sampling these 100,000 times, recalculating the binding in each pseudo-repeat, and finding the range in which 95% of these values fell.

Because larger molecules are more likely to be close to each other simply as a result of their size, the volume of the molecules needed to be determined to enable comparisons. This was conducted through a Monte Carlo method, in which 100,000 random coordinates within the periodic box were selected, and the fraction of these points within 5 Å of the molecule was calculated. In order to account for molecular flexibility, this was repeated at 150 randomly chosen simulation frames. The uncertainty presented is the 95% range of these volume fractions (Supplementary Tables S1–S3). The calculated volume fractions were determined to have a 96% correlation with empirical volume measurements for amino acids made by Tien et al., 2013 [87]. Expected time spent within the 5 Å threshold was observed to increase linearly with increasing molecular volume fraction, so artefactual “binding” resulting from larger molecular volumes was eliminated by dividing the proximity-based binding measure by the molecular volume (Supplementary Tables S7, S8, S12, S13, S17, S18, S22, and S23). This enabled comparison between the various systems. The preferred binding nucleotide for each amino acid was determined by ranking the size-adjusted proximity measure, and this was then compared to cognate nucleotide assignments in the genetic code, with a null hypothesis of uniform random preferences based on a binomial distribution. For overall elevation in preference, 200,000 randomised rank preferences of nucleotides to amino acids were generated, and the sum of these ranks was calculated for each randomised run. Additional assignments for hexacodonic amino acids were included. For amino acids with multiple cognate dinucleotides in the dinucleotide simulations, randomised preferences for the cognate dinucleotides were not allowed to be identical.

### 2.2. Hydrophobicity Trends

The influence of hydrophobicity was determined by using multiple linear regression. The proxy binding measures were compared against the volume of amino acids (before and after dividing by volume), the volume of nucleotides (again before and after dividing by volume), and hydrophobicity. Hydrophobicity was mainly determined using a composite scale, where an amino acid’s position was determined as the mean hydrophobicity rank of the amino acid across 43 hydrophobicity scales compiled by Trinquier and Sanejouand [8,80]. The individual scales were also compared on their own (Supplementary Tables S28 and S29). Regressions were performed in MATLAB R2020b using the “fitlm” function.

### 2.3. Rings

Where mentioned, instead of calculating the distance between all atoms, only the distance between the nitrogen atoms in the NMP rings and atoms in the amino acids were calculated. This is an imperfect measure, as nitrogen atoms are not homogenously laid out around the bases, but greatly simplified calculations due to irregular atom labelling produced by the software pipeline. This crude representation of the rings is flawed, but sufficient for some broad comparisons. Where considered, the volume of the rings was estimated using the same Monte Carlo method as for the full molecules, but using just the ring nitrogens.

### 2.4. NMR

Samples were created in HPLC Gradient Grade H_2_O. Monophosphate nucleotides were added at 0.1 mM, while amino acid concentrations varied from 0.1–100 mM. All samples were in 10 mM phosphate buffer with 0.6 mM MgCl_2_. The pH was adjusted to 7.40–7.42 using NaOH and HCl (measured with a Fisher Scientific accumet AE150 meter with a VWR semi-micro pH electrode) to mimic MD conditions. All chemicals were obtained from Fisher Chemicals and Sigma-Aldrich. If required, samples were stored in a 4 °C fridge. The samples were transferred to 5 mm diameter borosilicate glass NMR tubes for 600 MHz frequencies, with 10% (*v*/*v*) D_2_O as the lock signal and 0.001 (*w*/*v*) DSS as an internal chemical shift reference. Proton (1H) spectra were recorded at 298.2K on a Bruker Avance II 600 MHz spectrometer equipped with a TXO cryogenic probe.

The buffer solution was made using chemicals from Sigma-Aldrich (magnesium chloride anhydrous, ≥98%; potassium phosphate monobasic, 99.5–101%; potassium phosphate dibasic, 99.0–101%). All amino acids were purchased from Sigma-Aldrich, Gillingham, Dorset, UK, (Glycine, ≥99%; L-arginine monohydrochloride, ≥98%, L-aspartic acid sodium salt monohydrate, ≥98%; L-phenylalanine, 99%), as were all mononucleotides (adenosine 5′-monophosphate monohydrate, ≥97%; cytidine 5′-monophosphate, ≥99%; guanosine 5′-monophosphate, ≥99%; uridine 5′-monophosphate disodium salt, ≥99%). For pH adjustments, hydrochloric acid (Sigma-Aldrich, ≥37%) and sodium hydroxide (VWR Chemicals, Lutterworth, Leicestershire, UK, 98.5%) were used. All solutions, stocks, and samples were prepared in HPLC gradient-grade water from Fisher Chemicals, Loughborough, Leicestershire, UK.

Peak locations and other features were determined using Topspin v.4.1.4, and then binding parameters, including K_D_ and max shift, were inferred by fitting the results to the ligand binding equation (Equation (6)) from Williamson [88]. The following protons were used as probes: CMP H5 and H6, AMP H2 and H8, UMP H5, and GMP H8. In total, 500 fits were conducted using the “fit” function in MATLAB R2020b, with the following conditions: lower bounds of K_D_ = 0 M^−1^, delta shift max = 0 ppm; no upper bounds; starting point for delta shift max = 0 ppm, and the starting point for kD was randomly selected in the interval 0.5–1.5 M^−1^. In order to mitigate noise in the data, one datapoint was randomly excluded in each fit. The K_D_ was determined as the mean of these fits, with uncertainty as the range in which 95% of the fitted constants fell. The relative preferences for NMPs were further compared by randomly selecting inferred binding constants and ranking them 10,000 times.

## 3. Results

### 3.1. Amino Acids Prefer Cognate Nucleotides 

We simulated all 20 proteinogenic amino acids as zwitterions in the protonation state expected at pH 7, with mononucleotides in each of 3 charge states; −2, −1, and 0. Each simulation had a 10:1 ratio of amino acids to mononucleotides, as well as 150 mM MgCl_2_. The large number of amino acids increased the frequency of collisions in the simulations, strengthening the signal from subtle differences between the weakly interacting molecules. The systems were simulated for approximately 1.5 μs timescales. The charge state for dissolved mononucleotides at pH 7 should be −2, but we also used the −1 charge state, as that is more representative of what would be found in an RNA backbone (while lowering the combinatorial odds of interacting). Mononucleotides are unlikely to ever be in the neutral state under any relevant situations, but we performed these experiments anyway to better understand the effect of charge on the interactions.

**Figure 1:**
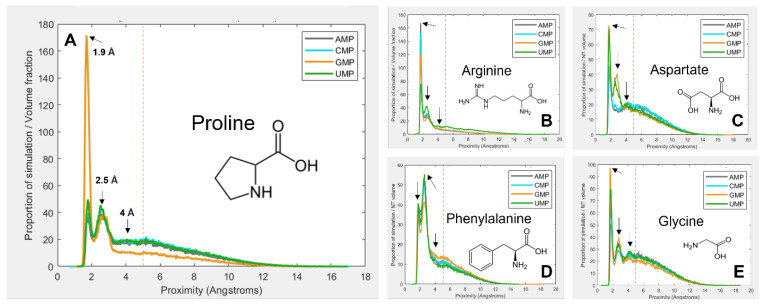
Volume-adjusted proximity probability distributions for a select different combinations of amino acids and nucleotides. Proximity is the closest atom of the nucleotide to the closest atom of the closest of 10 amino acids in the 40 Å periodic box. Proportion of simulation time is adjusted for the volume of the amino acid and nucleotide, so that different systems are comparable. Most interactions demonstrate multiple binding modes, at ~1.9 Å, 2.5 Å, and 4 Å. An additional peak at around 5.5 Å is also visible, which is interpreted as the average distance of the closest amino acid when not bound. The vertical red line indicates the 5 Å threshold for binding. (**A**) proline; (**B**) arginine; (**C**) aspartate; (**D**) phenylalanine; (**E**) glycine.

Figure 1 gives a selection of examples showing how the interactions vary between amino-acid–mononucleotide pairs, with mononucleotides in the −1 charge state. The figure demonstrates how preference for spending time at a given proximity varies. The proximity distribution is adjusted for molecular volume because larger molecules tend to be closer to one another simply due to the fact that they take up more of the simulation space. Figure 1A shows that proline spends a large proportion of simulation time bound to GMP at 1.9 Å, but is much less likely to be found at the same distance from the other 3 nucleotides. Another interaction mode is demonstrated at 2.5 Å, but proline behaves similarly with the 4 nucleotides here. There is a third interaction mode at 4 Å, but for proline, this is relatively indistinct. 

Figure 1B shows arginine, which demonstrates more subtle and complex differences in preferences among the four nucleotides. Figure 1C shows aspartate, which also shows relatively weak binding overall, but quite dramatic preferences for GMP and UMP at 2.5 Å. Figure 1D shows phenylalanine, which most commonly interacts at 2.5 Å rather than 1.9 Å. Finally, Figure 1E shows glycine, which interacts similarly with each of the 4 nucleotides, and demonstrates most clearly the 4 Å binding mode. Because of the wide range and complexity of the binding interactions, an agnostic approach was taken to determining relative preferences, whereby the total amount of time the molecules spent within 5 Å of each other was calculated (by integrating the area under the peaks within 5 Å). This threshold, as demonstrated by the vertical red dotted lines in Figure 1, aims to encompass all observed binding modes, while avoiding the ‘free-in-solution’ behaviour, where the closest unbound amino acid tends to be around 5.5 Å from the mononucleotide on average.

**Figure 2:**
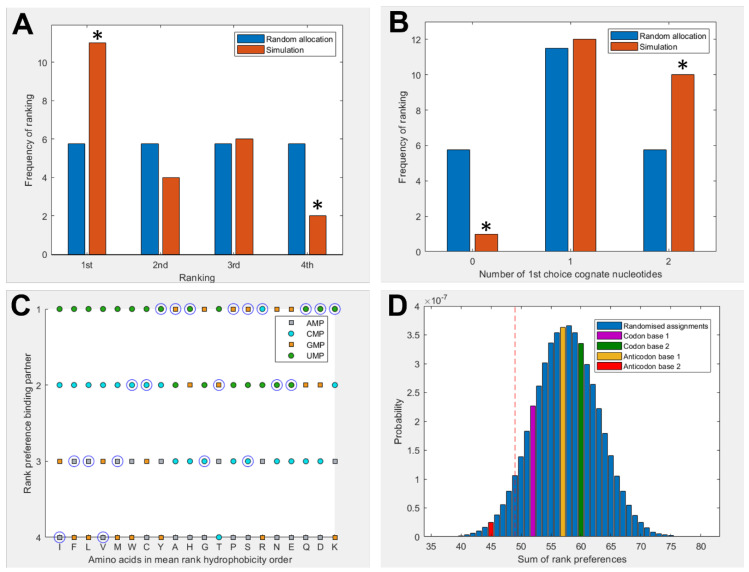
(**A**) Rank preference of the anticodonic middle-base nucleotide for each the 20 amino acids as predicted by MD simulations. Included are 3 additional cognate assignments for hexacodonic amino acids (arginine, serine, and leucine), giving 23 allocations. Random allocation shows equal chance of each amino acid interacting most strongly with any nucleotide. (**B**) Number of amino acids predicted to interact most strongly with both, 1, or no nucleotides cognate at the 1st or 2nd position of the anticodon or codon, compared with random allocations. (**C**) Rank preference of each amino acid for each nucleotide, ordered by mean hydrophobicity rank of amino acids [8,80]. The correct middle base for each amino acid is circled. The three hexacodonic amino acids are each circled twice, but for arginine and leucine, the two different codons share the same middle base, so the circles are overlaid (giving eleven correct predictions). Circular datapoints show pyrimidines; square datapoints are purines. Green is UMP, blue CMP, orange GMP, and grey AMP. (**D**) Sum of rank preferences of the amino acids for their cognate nucleotides compared to randomised preferences. Highlighted bars are scores for: codon base 1 (purple), codon base 2 (green), anticodon base 1 (yellow), anticodon base 2 (red). All nucleotides are in the −1 charge state in all panels, and interactions are given with respect to ring nitrogens. Asterisks indicate statistically significant deviation from the null distribution at the 5% level.

Figure 2 shows a summary of binding preferences among the full set of 20 amino acids and the 4 nucleotides in the −1 charge state. Binding preferences were calculated based on the proportion of simulation time spent within the 5 Å threshold, as shown in Figure 1. This measure was adjusted to account for amino acid and nucleotide volumes, and focuses specifically on interactions with the nucleobase. Included also were 3 additional pairings for amino acids with multiple cognate 1st and 2nd nucleotide base assignments. Figure 2A shows a significant elevation in amino acids that spend the greatest proportion of simulation time with their cognate anticodonic middle base [*p* = 0.0139] in the −1 charge state (which best matches the RNA backbone). This is the case for half of all amino acids (11 of 23 cognate nucleotides). There is a corresponding significant reduction in the number of amino acids that have the least favourable interactions with their anticodonic middle base [*p* = 0.0492]. Figure 2B shows that 95% of amino acids bound best to at least 1 cognate nucleotide in either their codon or anticodon (excluding base 3) [*p* = 0.0243] under these conditions; glycine was the only exception. We included the same three additional cognate codons/anticodons for the same reasons as in Figure 2A. However, no specific first choice preferences for cognate nucleotides in the −2 or neutral charge states were predicted using these measures (Supplementary Figures S2 and S3). 

Figure 2C breaks down these preferences more granularly, giving the ranked preferences of each amino acid by hydrophobicity. This reveals the prediction that hydrophilic amino acids are more likely to bind to the anticodonic middle base than to more hydrophobic amino acids. We can also see that UMP is predicted to be the most commonly preferred binding partner, which matches the observation from the modern codon table that UMP is the most common anticodonic middle base, utilised by 7 of 20 amino acids, and it is never redundant [80]. This trend was repeated in the neutral state, but did not appear in the −2 state (Supplementary Figure S3). Notably, AMP was predicted to be the least favoured binding partner; it was never ranked as first choice by any amino acids in either the −1 or neutral state (Figure 2C, Supplementary Figure S2), though this bias was not displayed in the −2 state. Given that AMP is the most hydrophobic nucleotide, this finding seems to indicate that our molecular dynamics simulations do not model hydrophobic interactions well.

Figure 2D compares the predicted preferences of the cognate middle bases to randomised preferences. For example, in the scenario where all amino acids spent the highest proportion of simulation time within 5 Å of their anticodonic middle-base nucleotide, the score for this base would be ranked 1 × 20 = 20. The worst-case scenario would be the cognate base being ranked 4 × 20 = 80. The 3 nucleotides with multiple codon assignments are also included, increasing the minimum possible score by 3. Left of the red dotted line represents the best 5% of randomised assignments. Compared to random assignments, cognate anticodonic middle bases were more strongly preferred than 99% of randomised pairings. An elevated preference was also observed for codon base 1, which gave a higher rank preference than 82% of randomised assignments. Elevations in affinity to codon base 1 nucleotides were also observed in the −2 state, with rank preferences greater than 95% of randomised assignments, but no elevation was observed for anticodonic nucleotides (Supplementary Figure S3). With nucleotides in the neutral charge state, the behaviour was indistinguishable from random (Supplementary Figure S2).

### 3.2. Hydrophobicity Plays a Role in Binding

**Figure 3:**
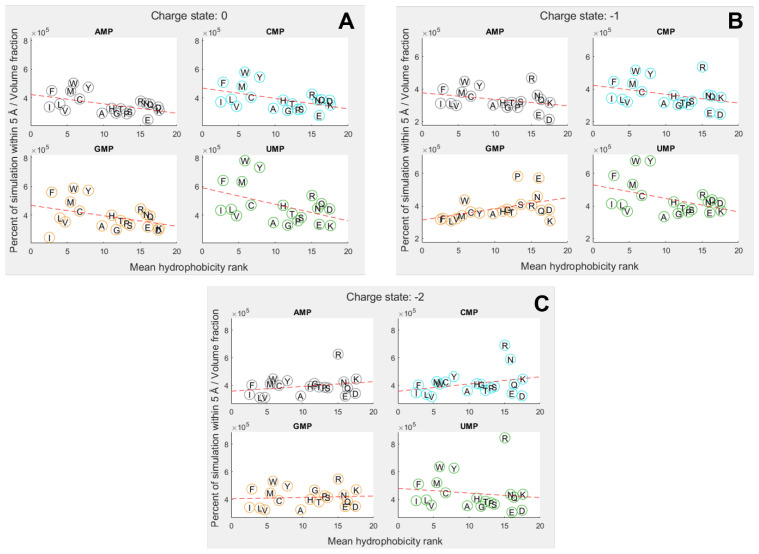
Proportion of simulation time for each amino acid within 5 Å of a nucleotide, adjusted for molecular volume. Amino acids are denoted by their single letter codes and organised by mean hydrophobicity rank, calculated from Trinquier’s 43 scales [8,80]. (**A**) phosphate charge state = 0; (**B**) phosphate charge state = −1; (**C**) phosphate charge state = −2. Dotted red line shows best fit from a linear regression. Distance is measured relative to nucleobase rings.

To explore how far amino-acid–nucleotide interactions were influenced by their relative hydrophobicity, we compared the interactions between the nucleobase rings against the hydrophobicity of the amino acids. We continued to use the volume-adjusted proximity measure. Due to the large variation in hydrophobicity given by different scales, we primarily utilised the mean of 43 scales collated by Trinquier and Sanejouand [8]. Figure 3 shows how the interactions were influenced by relative hydrophobicity in neutral, −1, and −2 charge states for each of the 4 nucleotides. The hydrophobicity rankings on the X-axis of Figure 3 are the same as those in Figure 2C, with the most hydrophobic amino acids being allocated the lowest numbers (at left), and the most hydrophilic with the highest numbers (at right). 

Overall, we found hydrophobicity to be a significant factor influencing binding in some cases, but not all (Supplementary Tables S24 and S25). In the neutral (Figure 3A) and −1 states (Figure 3B), the more hydrophobic amino acids tended to bind more strongly across the board, i.e., the slight negative correlation indicates that the more hydrophobic amino acids bound best. Yet, we expected to see the strongest inverse relationship with the most hydrophobic base (A), and that was not the case. Conversely, we expected the opposite relationship with uracil, the most hydrophilic base, but again that was not the case. GMP was the only exception in the −1 state. In this case, the positive correlation shows that the more hydrophobic amino acids bound less well than their hydrophilic counterparts. While G is sometimes considered to be a relatively hydrophobic base, the hydrophobicity of the bases is ambiguous, and we have followed Lacey et al. [15,89] in considering C to be more hydrophobic than G. 

In the −2 state (Figure 3C), the trends were less clear and not statistically significant at the 5% threshold. However, if the entire nucleotide was considered instead of just the rings, the −2 state showed a very strong negative dependence on hydrophobicity, meaning the most hydrophilic amino acids bound most strongly to all nucleotides (Supplementary Figure S4). A similar relative decrease in the binding of hydrophobic amino acids was observed for the other charge states when considering the whole mononucleotide, suggesting that the phosphate, especially when charged, was generally interacting with hydrophilic amino acids, whereas the rings were usually interacting with hydrophobic amino acids.

We also found that different specific hydrophobicity scales predicted different dependencies on hydrophobicity, but broadly, trends were similar across scales (Supplementary Tables S28 and S29). The strongest hydrophobicity dependence for ring interactions in the −1 state was predicted by the Krigbaum scale [90] (based on protein geometry) and the Sweet scale [91] (derived from mutational matrices) for the whole-molecule interactions. Krigbaum also predicted the strongest hydrophobicity dependence for the neutral state, but Sweet was once again the most predictive scale in the −2 state. This further suggests a complex interplay between ring and phosphate interactions, where the −1 state may balance both dynamics in these simulations.

### 3.3. NMR Corroborates Binding Interactions

**Figure 4:**
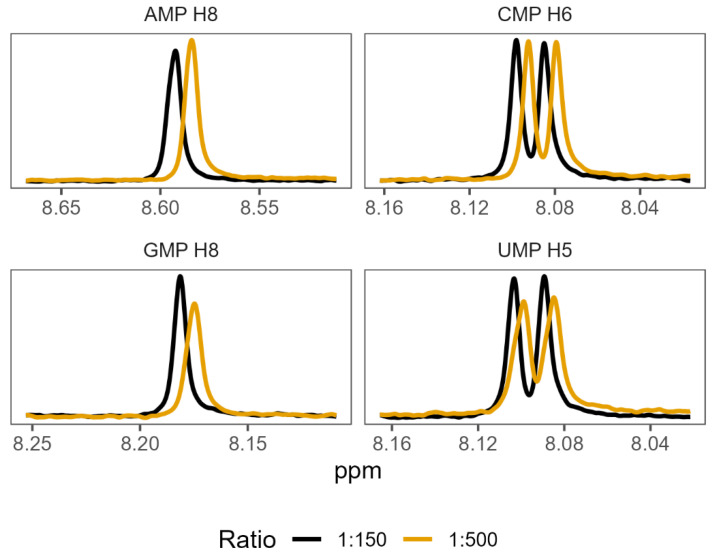
^1^H NMR spectra showing characteristic changes in proton chemical shift perturbations (or changes) as the ratio of amino acid to nucleotide increases. The panels show mixtures of phenylalanine with each of the 4 mononucleotides, at 2 representative ratios: 1:150 nucleotide to amino acid, and 1:500 nucleotide to amino acid. Numbers next to each NMP refer to the proton probes used for measurement of peak shifts (see Supplementary Figure S1 for numbering).

In order to validate the existence of preferential binding of the sort predicted by the molecular dynamics, we attempted to measure and compare the behaviour of a small selection of amino-acid–mononucleotide pairs using NMR. We chose to use phenylalanine, arginine, glycine, and aspartate, hoping to cover a variety of hydrophobicities and charges. We produced mixtures of amino acids and mononucleotides with 150 mM MgCl_2_ in potassium phosphate buffer at pH 7. We generated a range of ratios of amino acids to mononucleotides, maintaining the mononucleotides as a 0.1 mM concentration and varying the amino acid concentrations from 0.1 mM up to 100 mM. Increasing the concentration of a binding ligand, in this case the amino acids, should increase the proportion of the nucleotides in a bound complex. Effective binding changes the local environment of protons near the binding site, resulting in chemical shift perturbations (CSPs) of their peaks on the NMR spectra (Figure 4). The extent of CSPs will be modulated by varying amino acid concentrations, which allows us to infer details of the binding, such as the binding strength, K_D_ [88]. K_D_ measures the proportion of molecules in a complex for a given set of concentrations, where a lower K_D_ indicates that more molecules form a complex as a result of stronger binding.

Shifts in the proton peak location characteristic of binding interactions were identified in the overwhelming majority of amino-acid–mononucleotide pairs (Supplementary Tables S30–S33). While some systems appeared to reach maximum peak shift quickly (glycine and certain proton probes for aspartate), we were unable to reach saturation while retaining consistent conditions for certain pairings of phenylalanine and arginine. This supports the prediction of diverse and distinctive interactions from the molecular dynamics. We found that many of these systems were also very sensitive, producing noisy results at low concentrations. The inability to achieve binding saturation also produced large ranges for the inferred binding constants for some pairs. The varied structures of the different nucleobases also meant that proton probes are not distributed consistently (Supplementary Figure S1), making direct comparisons of binding challenging because not all binding sites have corresponding proton probes.

**Figure 5:**
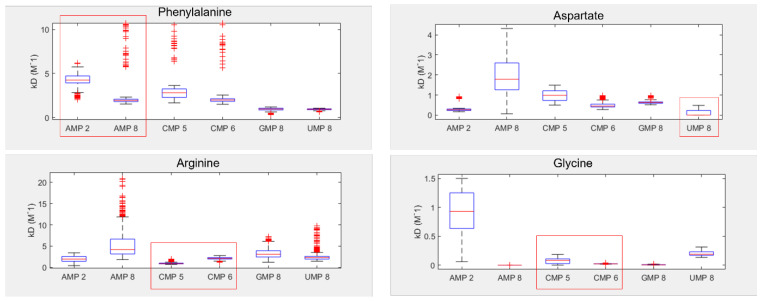
Inferred NMR binding constants (K_D_) for phenylalanine, aspartate, arginine, and glycine, with each of the four RNA bases. Lower K_D_ indicates stronger binding. Numbers next to each NMP refer to the proton probes used for measurement of peak shifts (see Supplementary Figure S1 for numbering). Red boxes highlight the cognate anticodonic middle bases for each amino acid.

Despite these issues, the results show that two of the four tested amino acids preferentially bound their anticodonic middle base (Figure 5), as determined by comparing the inferred K_d_ between curve fits. These were arginine, which bound best to CMP (the cognate anticodon) in 85% of fits, and aspartate, which bound best to UMP (the cognate anticodon) in 76% of fits. Aspartate also demonstrated elevated preference for AMP (the cognate codon) in 23% of fits. In contrast, phenylalanine demonstrated binding preferences for its codonic middle base instead, binding best to UMP (the cognate codon) in 60% of fits. AMP (the cognate anticodon) was actually its worst binding partner in 77% of fits. Glycine was the only amino acid that had no strong preference for either its codonic or anticodonic middle base, though GMP (the cognate codon) was its second best partner in 93% of fits. However, we note that glycine almost always interacted preferentially with AMP 8, whereas AMP 2 was typically the worst binding partner. AMP 8 is the proton next to the glycosidic nitrogen, while AMP 2 is the on the opposite side of the nucleobase (Supplementary Figure S1), suggesting that smaller amino acids might interact with only parts of larger nucleobase rings. This sort of regional preference may be important, as ignoring AMP 8 would make GMP (the cognate codon) the preferred binding partner in 95% of fits and CMP (cognate anticodon) the best in 5%, with this preference swapped for second best partner. Conversely, AMP 8 was a poor binding target for aspartate, whereas AMP 2 displayed relatively strong binding in this case. Similar variation appears to be present for CMP 5 vs. CMP 6, highlighting the wider problem of inconsistency in the proton probes between nucleotides.

Importantly, our NMR results follow similar behaviour to the molecular dynamic simulations on the microscale, wherein an elevated preference for the cognate nucleotides appears to be demonstrated. We note that the preference for the anticodonic middle base is predicted by both NMR and MD for arginine and aspartate, and that phenylalanine’s preference for UMP is repeated in both cases (even though this is not the cognate anticodon). Glycine’s lack of clear preference for cognate nucleotides is also predicted by both techniques. While our NMR investigations only analysed 20% of proteinogenic amino acids, we suspect that these patterns would continue to emerge over larger numbers of amino acids. Extending this experimental avenue is a goal for future research, although it will also be worth moving towards polynucleotides, which could allow for greater demonstrations of specificity and wider options for proton probes. 

### 3.4. Dinucleotides also Show Affinity for Their Cognate Amino Acids

**Figure 6:**
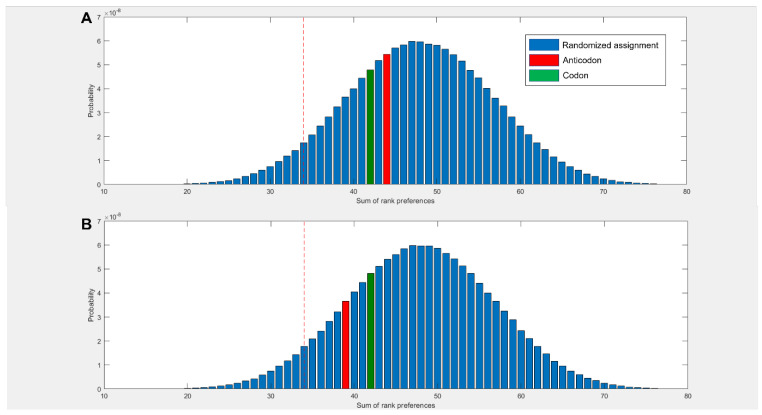
Sum of rank preferences for a selection of amino acids and dinucleotides with respect to proportion of simulation time spent within 5 Å, for (**A**) whole dinucleotide (**B**) ring nitrogens, adjusted for molecular volumes. If all amino acids had their cognate nucleotides as their best binding partner, this gives the lowest score. Red = bases 1 and 2, anticodonic dinucleotide. Green = bases 1 and 2, codonic dinucleotide. Left of red line is the top 5% of randomised assignments.

In order to take the first steps towards more complex systems, we simulated a selection (approximately 20%) of the 320 proteinogenic amino acid and dinucleotide pairs, using the same simulation and analysis pipelines as we did for the mononucleotides. This included 6 amino acids (Phe, Arg, Ser, Gly, Ala, and Asp) and 11 of the 16 dinucleotides, including all the codons and anticodons for the 6 amino acids, plus a selection of other dinucleotides, including homodimers and heterodimers (Supplementary Tables S19–S23). Figure 6 shows that these amino acids had weak elevations in preference for cognate dinucleotides. 

We observed that amino acids had a higher preference for their codonic nucleotides than about 73% of randomised assignments when considering either whole dinucleotides or the rings in isolation (Figure 6A,B). The amino acids had a higher affinity for their cognate anticodonic nucleotides than 65% of randomised assignments when considering the whole molecule (Figure 6A), which increased to 83% when considering the ring alone (Figure 6B). The previously observed high affinity for U was repeated with the dinucleotides—in this case, the dinucleotides with the highest average affinity across the six amino acids were UU, CU, and UC, in order of most to least favoured. Directionality effects of the sort predicted by Root-Bernstein [25,92] were also observed for a small subset of pairings, with differences in preferences predicted for glycine with AG vs. GA and with arginine and glycine for CG vs. GC. This was also observed for the rings in isolation, with differences between CG and GC for glycine, arginine, and phenylalanine, as well as between CU and UC for glycine and phenylalanine (Supplementary Tables S22 and S23).

## 4. Discussion

In this work, we have explored whether preferential stereochemical interactions between amino acids and nucleotides exist, and if so, whether the interactions match the patterns observed in the genetic code. Our results, using both MD and NMR, strongly support the hypothesis that stereochemical interactions do exist, and we find they often do match the modern codon assignments. The interactions are weak and probabilistic, but at scale they can be identified through the noise. Other notable features of the codon table, such as the frequency and non-redundance of uracil at anticodon base 2, and the patterning related to hydrophobicity, also appear to arise from these biophysical interactions. These patterns even hold true in modern codon reassignments, with 77% of known codon reassignments retaining the same middle base [93,94]. Taken together, our results corroborate Woese’s prescient conjectures from more than half a century ago, that the genetic code is based on a set of ancient and spontaneous interactions that have not been overwritten since the origin of life [1,11].

While this central idea is supported by both MD and NMR, some discrepancies remain. In NMR, all the nucleotides are likely to be in the −2 charge state at pH 7 [95], but the MD simulations mainly predicted cognate preferences in the −1 state (which matters because this corresponds to the charge state in the RNA backbone). Exact rank preferences were also not replicated perfectly between the two techniques. More generally, while our results support the hypothesis that hydrophobicity shapes interactions, we have struggled to identify patterns that directly resemble those observed in the codon table. For example, AMP being the most hydrophobic base [15,89], we expected it to interact most strongly with hydrophobic amino acids. UMP is the least hydrophobic base [15,89], so we expected it to interact most strongly with hydrophilic amino acids. We anticipated similar but weaker patterning for CMP and GMP, which are intermediate in their hydrophobicity. Yet, none of these differences were recovered in our simulations. We suspect that this discrepancy may reflect limitations in the fixed-charge forcefields used in our MD simulations, which also struggle to model dynamic charges and subtle changes in electron densities [96,97]. If so, then hydrophobic effects were poorly captured in our simulations, which could explain the poor overall binding of amino acids to AMP. 

This interpretation is supported by Figure 2, which shows that our simulations correctly predicted the cognate anticodon middle base for most hydrophilic amino acids (to the right end of the Trinquier scale), but fared badly with the hydrophobic amino acids (to the left). The negative charge on the phosphate group might then be an overpowering factor in our simulations, as suggested by the strong negative correlation between hydrophobicity and amino acid binding in the −2 state displayed by all nucleotides when interactions with the phosphate group are taken into consideration (Supplementary Figure S4). Viewed another way: as the phosphate charge becomes more negative, hydrophilic amino acids tend to bind for longer, while hydrophobic amino acids bind for shorter periods (Supplementary Figure S5). This effect is amplified slightly when interactions across the entire nucleotide are considered (Supplementary Figure S6), which suggests that the effect relates to charge, but not primarily interactions with the phosphate. Again, there were no obvious patterns in the interactions of bases depending on their own hydrophobicity. Overall, these correlations imply that our simulations were influenced by the charge environment, which partially obscured more subtle hydrophobic interactions between amino acids and bases in our MD simulations. 

While our NMR studies were not as extensive as our MD simulations, we found that NMR also predicted stronger anticodon interactions with hydrophilic amino acids (aspartate and arginine) than the hydrophobic phenylalanine (Figure 5). Working with hydrophobic amino acids and nucleotides was generally more troublesome due to their lower solubility, which gave wider ranges in predicted binding constants for these molecules. In our hands, then, both NMR and MD were better able to detect hydrophilic interactions, and neither method could predict preferences for the most hydrophobic base, adenosine, by amino acids coded by it at the middle base of the anticodon (U as the middle base of the codon). Because this is one of the strongest patterns in the code [8,9,80], if these hydrophobic interactions could be predicted better in the future, then the stereochemical preferences reported here for cognate anticodon interactions should become even stronger, making our conclusions conservative.

Another puzzle relates to the striking prediction that the anticodonic middle base and first codonic base were both preferred binding partners for cognate amino acids (Figure 2). This matches the observation that clear patterning is observed for both these bases in the codon table [80] and similar findings reported previously by Yarus et al. [21]. While the particular preferences for codon versus anticodon differ between our results and their 2005 study, that is not entirely surprising, as we were considering mononucleotides, while they were working with large RNA aptamers enriched in specific codons or anticodons. Critically, interactions with both cognate codons and anticodons shine through in both methods. This finding was repeated with the complementary prediction from Figure 6, which also showed an elevated affinity of amino acids for the cognate anticodon and codon dinucleotides. Taken together, these findings suggest the emergence of coding in some sort of binding pocket containing both the codon and anticodon. Nonetheless, it is still surprising that the binding affinities of amino acids to dinucleotides were not greater than those for mononucleotides. One possible explanation might be that very short polynucleotides are a known weak spot for MD [98]. Or, it could be that our results reflect a sampling bias due to the relatively limited number of amino-acid–dinucleotide pairs simulated. More interesting: if the patterns do reflect binding to a pocket, this would probably require the cognate nucleotides to be positioned opposite one another (as in normal codon–anticodon interactions). Without this multidirectional binding, the extra complexity of dinucleotides might confound preferences rather than strengthen specificity.

**Figure 7:**
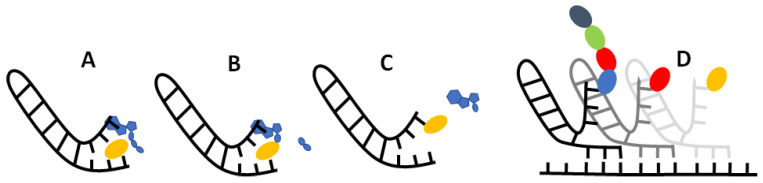
Possible mechanism for amino acid binding and adenylation on a proto-tRNA. (**A**) An amino acid (yellow oblong) binds to its cognate anticodon on a short RNA with a hairpin loop. An ATP (blue) stacks onto the terminal A adjacent to the anticodon. (**B**) Nucleophilic attack of the carboxylate oxygen on the α-phosphate releases the pyrophosphate tail (blue oblongs), which adenylates the amino acid. (**C**) Transfer of the amino acid from the adenosine to the 2′ ribose of the terminal A on the ‘acceptor stem’ results in an amino-acylated RNA. (**D**) Possible primordial mechanism of translation, where a flexible hinge of the proto-tRNA allows binding of the anticodon to a ‘codon’ on an adjacent proto-mRNA (where the reading frame is also determined by stereochemical interactions), enabling synthesis of short peptide sequences (multi-coloured adjacent oblongs) specified by the RNA sequence.

If our results do indeed point to some sort of selective RNA binding pocket for amino acids, then the challenge becomes: how could this pocket evolve into the modern translational and informational system? While much remains ambiguous, we imagine a model in which non-enzymatic chemistry and stereochemical interactions between amino acids and their cognate nucleotides could build incrementally towards the modern translational system. At issue here, once more, is Crick’s adaptor hypothesis [12,13], which stresses the absence of any direct correspondence between either the codon or anticodon and amino acid binding. On the contrary, on tRNA, the amino acid always binds to a CCA acceptor stem at one end of the molecule, while the anticodon–codon interactions take place far away at another end of the molecule. While there may be interactions between anticodons and amino acids in the ribosome [99], these have nothing to do with amino acid loading of the tRNA by aminoacyl tRNA synthetases. The question then becomes: how, physically, could the interaction between a binding pocket containing the anticodon and its cognate amino acid become separated into an interaction between a CCA acceptor stem and the cognate amino acid, and elsewhere, between the anticodon on tRNA and the codon on mRNA? We sketch a possible model in Figure 7.

We propose that translation began in autotrophically growing protocells, as outlined in the Introduction [63,80]. In this structured setting, the undirected polymerisation of nucleotides and amino acids could, in theory, occur spontaneously, driven by nucleoside triphosphates, notably, ATP [100], and catalysed by metal ions, such as Mg^2+^, and amino acids, such as aspartate (which is conserved in the active sites of modern RNA polymerases [101]) or lysine (which is conserved in the active site of modern RNA ligase enzymes [102]). Nucleotide polymerisation, in turn, should form short non-templated RNA aptamers, some of which may resemble a single hairpin loop of tRNA in structure [18,103], as depicted in Figure 7A. We imagine amino acids binding to these RNA pockets by way of the weak biophysical affinities demonstrated here, giving a statistical likelihood of repeatability. This biophysical patterning can, in principle, explain the emergence of information in biology. Consider: if random RNA sequences bind amino acids in a non-random fashion, and this facilitates the polymerisation of those amino acids into short templated peptides with non-random sequences, then biological meaning, linked with function, is introduced in the context of autotrophically growing protocells. Functions in growing protocells could include CO_2_ fixation, RNA polymerisation, and cofactor binding, all of which would facilitate protocell growth and heritability [63,80]. 

For RNA to template functional peptides, the next necessary step would be the transfer of the amino acid from its binding pocket onto the proto-tRNA acceptor stem. The universal acceptor stem is the CCA terminal of tRNA, and this is rigorously enforced in biology [104]. Curiously, CCA is an anticodon for glycine, the simplest amino acid with only an H for an R group, meaning that CCA is most likely to interact with the amino or carboxyl groups rather than the R group [105]. This general binding affinity means that the CCA could act as a universal ‘fishing rod’ for all amino acids. While we have not tested interactions with triplets, it is feasible that the presence of a CCA terminus could facilitate the binding of amino acids and may have begun to differentiate proto-tRNA from other sequences. In effect, any short RNA hairpin with a terminal CCA would behave like a proto-tRNA. The stacking of ATP on the terminal AMP of the CCA stem would help colocalise the ATP and amino acid, which is an essential function of amino acyl-tRNA synthetases (aaRS), as depicted in Figure 7A. Progenitors of aaRS, even very short polypeptides [18,106], have been shown to catalyse amino acid adenylation, and even tRNA acylation, by protecting the adenylate from water and colocalising reactants. Remarkably, modern tRNA can also self-load cognate amino acids (specifically, phenylalanine and methionine) at high pressure in the absence of aaRS [107]. We show the adenylation of an amino acid in Figure 7B, followed by transfer of the amino acid onto the CCA acceptor stem in Figure 7C, which acylates the tRNA. Thus, with no more than a short tRNA, we can picture the binding of an amino acid to a specific pocket, followed by its adenylation and acylation of the tRNA. These simple proto-tRNA molecules could eventually augment their specificity beyond the simple biophysical preferences shown here, for example, through the size discrimination of amino acids, which constitutes the deep split between the two classes of aaRS [108].

Exactly how such a tRNA could facilitate amino acid polymerisation is another question. As depicted in Figure 7D, we imagine that the anticodon was initially positioned at the opposite end of the tRNA hairpin loop to the CCA acceptor stem, on a flexible hinge that could twist around to interact directly with the codon on a proto-rRNA. In Figure 7D, we depict a small peptide growing from these interactions, but have not specified a mechanism. It is feasible that other short RNAs could catalyse amino acid polymerisation by colocalising proto-tRNA and mRNA templates. Such an assemblage of RNA would be the first steps to forming the proto-ribosome, and there is some evidence of this process in the structure and sequences of modern ribosomes [109,110]. These RNA complexes presumably catalysed peptide bond formation, but later, proto-ribosomes had to facilitate alignment with templates and begin to enforce reading frames from looser stereochemical roots. 

This model is admittedly based on some rather large extrapolations from the literature, but provides a route to build a full translational system from the simple biophysical interactions observed here. The critical steps to test will be the non-enzymatic chemistry of nucleotide polymerisation in water, the specificity of RNA pockets for amino acids, and the templated polymerisation of peptides on RNA. We will also address some of the mechanistic puzzles relating to the complexification of key components, notably, tRNA, which eventually moves the cognate nucleotides away from the acceptor stem. More mundane but immediate goals include exploring a wider range of experimental conditions for MD simulations. Advances in polarizable forcefields would also improve our results if they are able to simulate non-polar interactions with more sophistication. There is also plenty of scope for further work with NMR, including improving the precision of the experimentally determined binding constants, investigating more amino acids, and progressing to polynucleotides, though the number of possible combinations when using longer RNAs will present a challenge to comprehensive exploration. 

In conclusion, the results presented here support the existence of weak and probabilistic binding preferences between amino acids and nucleotides, as argued by Woese more than 50 years ago. Our results point to the origin of translation in binding pockets in RNA hairpin loops. The fact that these interactions are evident even with mononucleotides suggests that genetic information is based on spontaneous interactions built into the structure of the code from the very origins of polymerisation. That these biophysical interactions still shine through the genetic code shows they form a cornerstone that has supported the dazzling complexity of life ever since. 

## Data Availability

MD trajectories and NMR spectra are available from the authors upon request.

## Supplementary Materials

The following supporting information can be downloaded at https://www.mdpi.com/article/10.3390/life13051129/s1, Figure S1: The structure and labelling of nucleotide bases; Figure S2: Amino acid-nucleotide interactions with phosphate in the 0 charge state; Figure S3: Amino acid-nucleotide interactions with phosphate in the −2 charge state; Figure S4: Effects of amino acid hydrophobicity on interactions with the whole nucleotide; Figure S5: Effects of phosphate charge on amino acid interactions with nucleotide rings; Figure S6: Effects of phosphate charge on amino acid interactions with the whole nucleotide; Tables S1–S3: Volumes of molecules; Tables S4–S8: Interaction data from the 0 charge state; Tables S9–S13: Interaction data from the −1 charge state; Tables S14–S18: Interaction data from the −2 charge state; Tables S19–S23: Interaction data with dinucleotides; Tables S24–S27: Regressions of interactions and hydrophobicity; Tables S28–S31: Regressions of interactions and further hydrophobicity scales; Tables S32–S36: Binding constants and comparisons of NMR data; Tables S37–S41: NMR peaks and shifts data.
